# Risk factors for virological failure and subtherapeutic antiretroviral drug concentrations in HIV-positive adults treated in rural northwestern Uganda

**DOI:** 10.1186/1471-2334-9-81

**Published:** 2009-06-03

**Authors:** Laurence Ahoua, Gunar Guenther, Loretxu Pinoges, Paul Anguzu, Marie-Laure Chaix, Clotilde Le Tiec, Suna Balkan, David Olson, Charles Olaro, Mar Pujades-Rodríguez

**Affiliations:** 1 HIV/AIDS Department, Epicentre, Paris, France; 2 Medical Department, Médecins Sans Frontières, Arua, Uganda; 3 Laboratory of Virology, Paris Descartes University, Paris, France; 4 Laboratory of Toxicology, Bicêtre Hospital, Kremlin Bicêtre, France; 5 Medical Department, Médecins Sans Frontières, Paris, France; 6 Medical and Administrative Hospital Direction, Arua Regional Hospital, Arua, Uganda; 7 International and Environmental Health, Institute of Social and Preventive Medicine, Bern, Switzerland

## Abstract

**Background:**

Little is known about immunovirological treatment outcomes and adherence in HIV/AIDS patients on antiretroviral therapy (ART) treated using a simplified management approach in rural areas of developing countries, or about the main factors influencing those outcomes in clinical practice.

**Methods:**

Cross-sectional immunovirological, pharmacological, and adherence outcomes were evaluated in all patients alive and on fixed-dose ART combinations for 24 months, and in a random sample of those treated for 12 months. Risk factors for virological failure (>1,000 copies/ml) and subtherapeutic antiretroviral (ARV) concentrations were investigated with multiple logistic regression.

**Results:**

At 12 and 24 months of ART, 72% (n = 701) and 70% (n = 369) of patients, respectively, were alive and in care. About 8% and 38% of patients, respectively, were diagnosed with immunological failure; and 75% and 72% of patients, respectively, had undetectable HIV RNA (<400 copies/ml). Risk factors for virological failure (>1,000 copies/ml) were poor adherence, tuberculosis diagnosed after ART initiation, subtherapeutic NNRTI concentrations, general clinical symptoms, and lower weight than at baseline. About 14% of patients had low ARV plasma concentrations. Digestive symptoms and poor adherence to ART were risk factors for low ARV plasma concentrations.

**Conclusion:**

Efforts to improve both access to care and patient management to achieve better immunological and virological outcomes on ART are necessary to maximize the duration of first-line therapy.

## Background

In 2007, approximately 810,000 adults were living with HIV/AIDS in Uganda, and 25–49% of those requiring antiretroviral therapy (ART) were receiving treatment [1]. Studies conducted in urban areas of Uganda have shown good therapeutic outcomes in HIV-infected adults treated with ART [2-7]. However, little is known about immunovirological treatment outcomes, likelihood of achieving therapeutic plasmatic concentrations of antiretroviral (ARV) drugs, and adherence to treatment of patients treated using a simplified management approach [8] in rural areas of developing countries. Also, risk factors for virological failure have not been extensively investigated.

Rural programs face numerous challenges concerning HIV care and treatment in Africa, including lack of appropriate tools to diagnose common opportunistic infections and qualified human resources. The scale-up of ART in recent years has not necessarily been accompanied by an increased strengthening of health systems, and this disparity is likely to negatively affect the quality of care in HIV/AIDS programs.

The main objective of this study was to describe clinical and immunovirological response to ART, patients' self-reported adherence, and plasma levels of ARV in HIV-infected adults who received free World Health Organization (WHO)-recommended ARV drug regimens as fixed-dose combinations (FDCs) for 12 or 24 months in Arua, a rural area in northern Uganda. Furthermore, we investigated potential risk factors for virological failure and for subtherapeutic concentrations of non-nucleoside reverse transcriptase inhibitors (NNRTI) or protease inhibitors (PI).

## Methods

### Study population

Since 2002, Médecins Sans Frontières (MSF) in collaboration with the Ugandan Ministry of Health has provided ART free of charge in the regional referral hospital of Arua. The program initially served the whole West Nile region, but decentralization of patients on ART to other district and missionary hospitals was started in 2004. Eligibility criteria for ART are those recommended in the WHO guidelines for scaling up ART in resource-poor settings [9]. Patients are seen by nurses monthly or every 3 months for drug refills and to assess ARV drug toxicity; and by clinical officers every 2 or 6 months. CD4 T-cell counts are currently monitored at ART start (for patients with no WHO clinical stage 4 conditions) and every 6 months (yearly after the first year on ART since 2005). No routine viral load (VL) monitoring is performed.

Data routinely collected using standardized forms and entered into the FUCHIA software (Epicentre, Paris) were, at program inclusion, date of birth or age, and history of ARV use; and at ART start, date, regimen, clinical WHO conditions diagnosed, weight, height, and CD4 T-cell counts. By November 2005, approximately 8,000 patients were followed in the program, 90% of them were adults, and 45% were alive and followed on ART.

### Study design

Before the start of the study (November 2005), we identified patients included in two observational open cohorts of HIV-infected patients who initiated ART in the previous 12 ± 2 months (M12 cohort) and 24 ± 2 months (M24 cohort). Patients found to be alive and still on ART were eligible for participation in a cross-sectional survey if they were contacted within the study window period, were non-pregnant or breastfeeding at the time of the study, and provided written informed consent. The study sample size was calculated assuming that 16% (M12) and 20% (M24) of these patients, respectively, would have detectable VL [10,11]. Hence, we included all patients from the M24 cohort (N = 369) and randomly selected 275 patients from the M12 cohort (N = 701).

### Data collection and laboratory testing

During the cross-sectional survey conducted between 28^th ^November 2005 and 2^nd ^May 2006, we used a standardized questionnaire to collect information on diagnosed acute severe illnesses, ARV-related toxicity, anthropometric measurements, and adherence to treatment. Two self-reported indicators of adherence were calculated: the percentage of pills taken in the last 4 days (number of pills taken as a proportion of the total number of pills prescribed); and the percentage of reported adherence in the last 30 days using a 6-point visual analogue scale (30-day VAS). Good adherence was defined as a value ≥ 95% pills or a VAS score >4 [9].

Plasma concentrations of nevirapine (NVP), efavirenz (EFV), and nelfinavir (NFV) were determined by high-performance liquid chromatography [12,13] on blood samples collected 12 hours after ingestion of the last dose of ARV. Hemoglobin, full blood cell count, alanine aminotransferase, and plasmatic creatinine concentrations were measured. CD4 T-cell counts were quantified using semi-automated (Cyflow counter, Partec, Münster, Germany) or manual (Dynabead, Dynal Biotech SA, Compiègne, France) techniques. HIV-1 RNA was quantified with the ANRS generic real-time PCR test [14]. Genotypic resistance tests were performed in plasma samples with HIV-1 RNA ≥ 1,000 copies/ml using the consensus technique of the AC11 ANRS 2007 v16; possible resistance was considered as resistance [15,16].

### Statistical analysis

#### Retrospective cohort analysis

Probabilities of survival for all patients included in the two cohorts were estimated using Kaplan-Meier methods. Patients missing their last clinical appointment for 2 months or more were considered lost to follow-up (LTFU).

#### Analysis of cross-sectional survey data

Characteristics of patients, ARV toxicity, and adherence were described using standard statistics. Laboratory ARV toxicity was graded according to the 2004 AIDS Clinical Trials Group criteria [17]. We defined immunological failure as a CD4 T-cell measurement of ≤ 100/mm^3 ^at 12 months, ≤ 200/mm^3 ^at 24 months, or below the value recorded at ART start at 12 or 24 months of ART [18,19]. Virological failure was defined as the presence of ≥ 1,000 HIV RNA copies/ml. Characteristics of patients at ART start and at the time of the survey, ARV toxicity, ARV plasma levels, and adherence were described using standard statistics.

Based on ARV therapeutic ranges, (4,000–8,000 ng/ml for NVP, 1,000–4,000 ng/ml for EFV, and 1,000–4,000 ng/ml for NFV [20]), we defined 3 levels of plasma drug concentrations: low (below the lower therapeutic range limit), normal (within the therapeutic range), and high (above the higher therapeutic ranged limit).

We investigated predictive factors for virological failure (HIV-1 RNA ≥ 1,000 copies/ml) and low plasma NVP, EFV, or NFV concentrations using a multiple logistic regression analysis. The covariates studied were sex, age (15–34 vs. ≥ 35 years), level of education (primary or no education vs. secondary or higher), ARV-experienced, WHO staging (1/2 vs. 3/4), tuberculosis (TB) diagnosis after ART initiation, baseline or nadir CD4 cell count (<50 vs. ≥ 50 cells/mm^3^), weight ≤ baseline, cohort (M12 vs. M24), presence of ARV-related toxicity, and poor adherence to ART (95% vs. ≥ 95%). For each outcome, we applied a backwards elimination approach to an initial model including those covariates associated with the outcome in univariate analyses (*P *< 0.25). The goodness-of-fit χ^2 ^test was used to assess the fit of the final models [21]. All analyses were performed in Stata 9.0 (Stata Corp., College Station, TX, USA).

The protocol was approved by the Uganda National Council for Science and Technology and the Ugandan AIDS Research Committee.

## Results

### Retrospective cohort analysis

By the end of the study (May 2006), 130 patients of the 1,523 patients included in the studied cohorts (48/967 of M12 and 82/556 of M24) had died, and 282 (187 of M12 and 95 of M24) had been lost-to-follow-up. Eighty-three percent of the 130 recorded deaths occurred during the first 6 months of ART. Probabilities of survival at 12 and 24 months of ART were 0.91 (95% CI 0.89–0.92) and 0.88 (95% CI 0.86–0.90), respectively, and probabilities of remaining in care were 0.76 (95% CI 0.74–0.78) and 0.70 (95% CI 0.67–0.72), respectively.

### Participants in the cross-sectional survey

**Figure 1:**
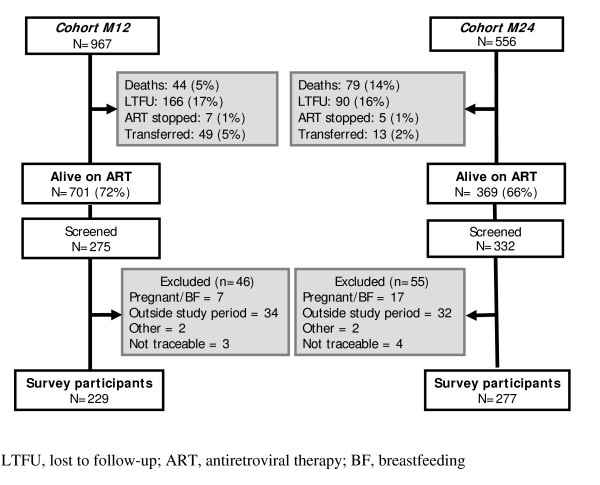
**Patient cohort profiles at study start (November 2005)**.

Of the 967 adults who initiated ART between September 2004 and July 2005 (M12 cohort), 701 (72%) were still alive and on ART at the time of the start of the survey. Of the 556 adults who initiated ART between September 2003 and July 2004 (M24 cohort), 369 (66%) were alive and on ART. A total of 229 (83% of eligible) M12 and 277 (75% of eligible) M24 patients participated in the cross-sectional survey. Reasons for exclusion were pregnancy or breastfeeding, inability to trace the patient, or a screening visit outside the study period (12 ± 2 or 24 ± 2 months of ART) (Figure 1). Men and patients ARV-experienced at ART start were more likely to be included in the study than women or ARV-naïve patients.

### Survey participant characteristics at ART start

Study patients had been followed in the HIV program of Arua prior to ART initiation for a median time of 6 (M12) and 4 (M24) months, and 98% were ARV-naïve. At ART initiation, median age was 37 years, 88% of patients were in WHO clinical stage 3 or 4, and approximately 17% had a body mass index (BMI) <17 kg/m^2 ^(Table 1). More than 25% had a CD4 cell count <50 cells/mm^3^, and most patients received stavudine/lamivudine/NVP (d4T/3TC/NVP). Nine percent of patients had been diagnosed with TB.

**Table 1:** Patient characteristics and outcomes

Patient characteristics	Cohort M12 (N = 229)	Cohort M24 (N = 277)
**Characteristics at ART start**		
Women (%)	151 (65.9)	173 (62.4)
Median age, years [IQR]	36.8 [32.0–42.3]	36.5 [30.8–42.6]
ARV naïve (%)*	225 (98.2)	273 (98.6)
Median follow-up before ART, months [IQR]	6.2 [3.9–11.4]	3.7 [1.8–7.7]
First ART regimen prescribed d4T/3TC/NVP	226 (98.7)	265 (95.7)
WHO stage 3 or 4 (%)	201 (87.7)	246 (88.8)
Body mass index <17 kg/m^2 ^(%)	38 (17.8); n = 214	43 (16.8); n = 256
CD4 cell count, cells/mm^3^	n = 133	n = 239
Median [IQR]	100 [45–147]	93 [40–144]
<50 (%)	36 (27.1)	73 (30.5)

**Characteristics at time of survey**		
Non-cumulative WHO stage at survey (%)	n = 223	n = 267
Asymptomatic	136 (61.0)	160 (59.9)
Stage 1 or 2	63 (28.2)	86 (32.2)
Stage 3 or 4	24 (10.8)	21 (7.9)
Body mass index, kg/m^2^		
Median [IQR]	20.4 [18.9–22.0]	20.8 [19.0–22.5]
<18.5 kg/m2 (%)	43 (18.8)	52 (18.7)
Median weight gain since ART start, kg [IQR]	3 [1–6]	2 [0–7]

ARV-clinical related symptoms (%)		
Asthenia	70 (30.7)	84 (30.3)
Digestive disorders	132 (57.6)	146 (52.7)
Neurological disorders	102 (44.5)	111 (40.1)
Dermatological disorders	70 (30.6)	84 (30.3)
Morphological disorders	39 (17.0)	72 (26.0)

CD4 cell count, cells/mm^3^		
Median [IQR]	223 [163–308]	238 [172–321]
<200 (%)	92 (40.2)	100 (36.1)
Median gain since ART start [IQR]	+115 [59–170]	+139 [75–215]

Pills taken in last 4 days (%)		
Good adherence	200 (87.3)	258 (93.1)
Poor adherence	29 (12.6)	19 (6.9)
30-day VAS (%)		
Good adherence	198 (86.5)	250 (90.3)
Poor adherence	31 (13.5)	27 (9.7)

Plasmatic NVP level (%)	n = 204	n = 258
Low	39 (19.1)	32 (12.4)
Normal	115 (56.4)	172 (66.7)
High	50 (24.5)	54 (20.9)
Plasmatic EFV level (%)	n = 23	n = 18
Low	1 (4.4)	-
Normal	13 (56.5)	11 (61.1)
High	9 (39.1)	7 (38.9)

VAS, visual analogue scale; IQR, interquartile range

*ARV-naïve does not include women who received PMTCT prophylaxis.

### Patient characteristics at survey

At the time of the survey, 10 patients were treated for active TB, and 86% of patients remained on FDC d4T/3TC/NVP. No patients were receiving second-line therapy. All participants reported at least one type of clinical intolerance, most frequently headache and digestive symptoms such as epigastric/abdominal pain or meteorism. Peripheral polyneuropathy and dermatologic disorders were reported in more than 30% of patients in both cohorts. Forty percent of patients had at least one moderate or mild abnormal laboratory result. Seven patients had grade 3 or 4 abnormal laboratory results, 6 had grade 3 neutropenia (3 M12 and 3 M24), and 1 (M24) grade 4 anemia.

### Treatment adherence

In the M12 cohort, good adherence to ART, defined as all pills taken in the last 4 days, was reported by 200 (87.3%) patients (or by 198 [86.5%] patients when the 30-day VAS was used). In the M24 cohort, good adherence to ART in the last 4 days was reported by 258 (93.1%) patients (250 [90.3%] using VAS) (Table 1).

Overall, the two measures of adherence were moderately correlated (Pearson's coefficient 0.48) and correlation was stronger for M12 than for M24 measurements (0.59 and 0.35, respectively). Twenty-seven (6.0%) patients considered as having good adherence by VAS reported to have forgotten taking one or more pills in the last 4 days; while 37 (8.1%) of those considered as having good adherence through 4-day recall reported poor adherence with VAS. A total of 85 (16.8%) patients were categorized as poorly adherent to ART by any of the two methods.

### Immunological and virological failure

At M12, median CD4 cell count was 223 cell/mm^3^, immunological failure was diagnosed in 7.9% of patients, and 40% had <200 cells/mm^3 ^(Table 1). Furthermore, 75% (171/229) of patients had undetectable HIV RNA (<400 copies/ml), and 89.1% (204/229) had <1,000 HIV RNA copies/ml.

At M24, median CD4 cell count was 238 cells/mm^3^, immunological failure was diagnosed in 38% of patients, and 36% of patients had <200 cells/mm^3^. Seventy-two percent of patients (198/277) had HIV viral suppression, and 81.9% (227/277) had <1,000 HIV RNA copies/ml.

### Frequency of drug-resistant viruses

We performed genotypic tests for 62 (82.7%) of patients with VL ≥ 1,000 copies/ml (PCR amplification failed for 13 samples) (Table 2). Five patients (8%) had no mutations. Wild-type viruses were found in 16 (26%; 6 at M12 and 10 at M24) patients. Fourteen (70%) patients at M12 and 32 (76%) at M24 had ≥ 1 mutation conferring drug resistance to NRTIs or NNRTIs. The overall frequency of resistant virus among treated patients was 7.3% (14/191) at M12 and 13.3% (32/240) at M24 (excluding 62 patients with VL = 400–999 and 13 with failing amplification reaction); or 18.8% (43/229 and 52/277, respectively) if the 75 excluded patients were assumed to have resistance mutations.

**Table 2:** Results of virological and genotypic resistance testing by cohort

	Cohort M12	Cohort M24
HIV RNA ≥ 1,000 copies/ml (%)	25	50

Available genotypic resistance (%)	20 (80.0)	42 (84.0)

Wild-type virus (%)	6 (30.0)	10 (23.8)
Resistance to ≥ 1 ARV drug (%)	14 (70.0)	32 (76.2)
Resistance to EFV and NVP (%)	3 (15.0)	1 (2.4)
Resistance to 3TC, EFV, and NVP (%)	9 (45.0)	22 (52.4)
Extensive resistance* (%)	2 (10.0)	9 (21.4)

ARV, antiretroviral; EFV, efavirenz; NVP, nevirapine; 3TC, lamivudine

*Resistance to EFV, NVP, 3TC, stavudine, zidovudine, or tenofovir, and didanosine, or multidrug resistance.

The most prevalent NRTI and NNRTI mutations were M184V and K103N, respectively. In the M12 cohort, 3 viruses were resistant to EFV and NVP, and 9 to both 3TC and NNRTIs (EFV and NVP). One virus was resistant to 3TC, d4T, zidovudine (AZT), and NNRTIs (EFV and NVP), and 1 harbored K65R, K103N, Y181C, and G190A mutations conferring resistance to tenofovir (TDF), EFV, NVP, and possibly didanosine (ddI).

In the M24 cohort, only 1 virus was resistant to NVP and EFV, and 22 to both 3TC and NNRTIs. Six viruses with resistance mutations to both 3TC and NVP had additional resistance to d4T and/or AZT. Three viruses harbored a K65R mutation conferring resistance to TDF and possibly to ddI. One virus had a multidrug resistance profile (K65R, T69A, V75I, K103N, F116Y, Q151M, V179S, Y181C, and M184V), conferring resistance to all the NRTIs and to EFV and NVP.

Thymidine analogue mutations (TAMs) conferring resistance to AZT and d4T were observed in 5% and 14% patients at M24, respectively. Resistance to both TDF and ddI were described in 5% (1/20) at M12 and 7% (3/42) at M24. The NRTI multidrug resistance complex (Q151M) was found in 1 patient at M24. None of the viruses had mutations conferring resistance to the new NNRTIs (etravirine). Seven patients were resistant to AZT, d4T, 3TC, NVP, and EFV (6 in M24).

### Factors associated with virological failure

Factors significantly associated with virological failure were longer time on therapy (OR 2.0; 95% CI 1.2–3.6 for M24 compared with M12 patients), ≥ 35 years of age at ART start (OR 0.5; 95% CI 0.3–0.8), TB diagnosis during ART (OR 2.3; 95% CI 1.0–5.0), weight less than baseline weight at time of ART initiation (OR 2.2; 95% CI 1.3–3.9), poor adherence (using VAS, OR 3.9; 95% CI 1.9–7.7), low NNRTI plasma concentration (OR 2.4; 95% CI 1.2–4.8), and presentation of general signs or symptoms (myalgia, hepatomegaly, splenomegaly, or lymphadenopathy; OR 2.6; 95% CI 1.5–4.5) (Table 3).

**Table 3:** Factors associated with virological failure and low plasma NNRTI concentrations

	Virological failure					Low NNRTI concentrations				
	
	n/N	Univariate analysis		Multivariate analysis^†^		n/N	Univariate analysis		Multivariate analysis^†^	
	
		OR (95% CI)	*P *value	OR (95% CI)	*P *value		OR (95% CI)	*P *value	OR (95% CI)	*P *value
**M24 cohort **(vs. M12)	50/506	1.8 (1.1–3.0)	0.02	2.0 (1.2–3.6)	0.01	32/505	0.6 (0.4–1.0)	0.06	0.7 (0.4–1.1)	0.15

**Age at ART start **(vs. 15–34)										
≥ 35 years	40/506	0.5 (0.3–0.9)	0.02	0.5 (0.3–0.8)	0.01	46/505	1.1 (0.7–1.9)	0.58	-	-

**School level **(vs. secondary or higher)										
Primary or no education	47/506	0.8 (0.5–1.4)	0.50	-	-	53/505	1.5 (0.9–2.6)	0.14	1.3 (0.8–2.4)	0.3

**Non-cumulative WHO stage at survey **(vs. asymptomatic)										
Stage 1–2	32/490	1.9 (1.2–3.3)	0.04	1.9 (1.1–3.3)	0.06					
Stage 3–4	6/490	1.1 (0.4–2.8)		0.8 (0.3–2.2)		-	-	-	-	-
**TB episode after ART**	11/506	2.1 (0.9–4.3)	0.06	2.3 (1.0–5.0)	0.04	-	-	-	-	-
**Weight **≤ baseline	32/506	1.9 (1.2–3.3)	0.009	2.2 (1.3–3.9)	0.004	15/505	0.6 (0.3–1.1)	0.07	0.6 (0.3–1.1)	0.11

**ARV-related toxicity**										
Digestive	49/506	1.6 (0.9–2.8)	0.05	1.5 (0.8–2.6)	0.15	48/505	1.8 (1.1–3.0)	0.03	1.7 (0.9–2.9)	0.05
Dermatologic	31/506	1.8 (1.1–2.9)	0.03	1.6 (0.9–2.8)	0.08	21/505	0.9 (0.5–1.6)	0.82	-	-
General symptoms*	30/506	2.3 (1.4–3.9)	0.002	2.6 (1.5–4.5)	0.001	17/505	0.9 (0.5–1.6)	0.74	-	-
**Poor adherence to ART **(<95% vs. ≥ 95%)										
% pills taken in last 4d	11/506	1.8 (0.0–3.8)	0.11	1.1 (0.4–2.6)	0.85	17/505	4.0 (2.1–7.7)	0.0001	3.8 (1.9–7.4)	<0.0001
30-day VAS	19/506	3.4 (1.8–6.4)	0.0002	3.9 (1.9–7.7)	0.0001	-	-	-	-	-

**Nadir CD4^+ ^<50 cells/mm^3 ^**(vs. ≥ 50)	24/506	1.5 (0.9–2.6)	0.14	1.5 (0.8–2.6)	0.20	19/503	1.1 (0.6–1.9)	0.77	-	-

**Low NNRTI plasma concentration at survey**	17/505	1.9 (1.1–3.7)	0.03	2.4 (1.2–4.8)	0.01	-	-	-	-	-

TB, tuberculosis; VAS, visual analogue scale; NNRTI, non-nucleoside reverse transcriptase inhibitor; OR, odds ratio; CI, confidence interval.

*General signs or symptoms: myalgia, hepatomegaly, splenomegaly, or lymphadenopathy; VAS.

^†^Multiple logistic regression using backward elimination approach and controlling for the effect of all variables for which adjusted estimates are shown.

### NNRTI plasma concentrations

In the M12 cohort, plasmatic concentrations of NVP and EFV were low in 19.1% (39/204) and 4.3% (1/23) of patients, respectively, and high in 24.5% (50/204) and 39.1% (9/23), respectively. NFV plasmatic concentrations were within normal therapeutic ranges (n = 2). In the M24 cohort, low plasma NVP concentrations were observed in 12.4% (32/258) of patients and high NVP and EFV concentrations in 20.9% (54/258) and 38.9% (7/18), respectively.

### Factors associated with low NNRTI plasma concentrations

**Figure 2:**
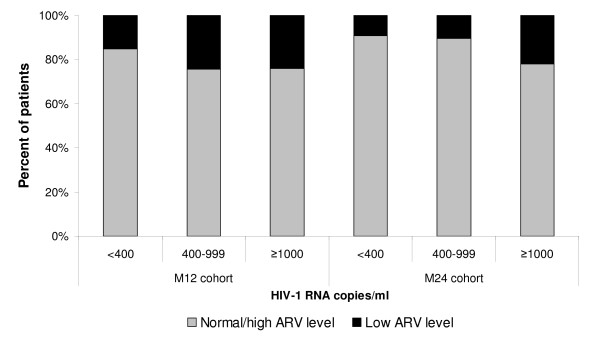
**HIV-1 RNA values by ARV drug plasma level and by cohort**.

Overall, the proportion of patients with low ARV concentrations significantly increased with increasing HIV RNA levels (from 12.8% in patients with undetectable VL to 22.7% in those with virological failure, *P *= 0.02). This association was observed in both cohorts (Figure 2).

Poor adherence in the last 4 days (OR 3.8, 95% CI 1.9–7.4) and reported digestive disorders (OR 1.7, 95% CI 0.9–2.9) were significantly associated with the presence of low plasma NNRTI or NFV concentrations (Table 3), but these factors were unrelated to the weight of the patients (data not shown).

## Discussion

In the HIV care and treatment program of Arua, in rural Uganda, more than 70% of the patients remained in care after 1 or 2 years of treatment, but over 35% of patients remained at risk of developing opportunistic infections (<200 cells/mm^2^). Estimates of retention in care appear to be more favorable than those reported during the early years of the Drug Access Initiative (DAI) program among individuals treated in urban hospitals of Kampala, where patients were requested to pay the care fees (49–56% at 1 year, and 46% at 2 years of ART) and more than 30% were ARV-experienced [3,5]. Like in other African settings, most of our patients started treatment at an advanced stage of HIV disease [4,10,22-24], which partly explains why a considerable proportion of patients had low CD4 cell counts after 1 or 2 years of therapy. As reported previously [11,25-27], early mortality in our cohorts was high with 88% of deaths occurring in the first 6 months of follow-up under ART. Delays in access to care (>80% of patients started treatment at an advanced clinical stage of HIV disease), delayed diagnosis and treatment of severe opportunistic infections (e.g. TB), undiagnosed severe immune reconstitution inflammatory syndromes, and nutritional deficiencies might have contributed to this increased early mortality [28-31].

Despite the frequency of clinical intolerances reported, the initial ART regimen was well tolerated, and few cases of severe (grades 3 or 4) laboratory-based toxicity were diagnosed. Self-reported adherence at 12 and 24 months of ART was high and similar to that reported in other studies, both in resource-limited countries [32] and North America [33,34], with a higher proportion of patients reporting good adherence in the M24 cohort than the M12 cohort (90% compared with 80%, respectively). Although reported adherence is subject to measurement error and we cannot exclude that some patients over-reported their level of adherence to ART during the survey, these findings were consistent with plasmatic ARV pharmacological measurements obtained in the same patients. However, the reported levels of adherence do not necessarily reflect patient's compliance throughout the duration of treatment.

The observed immune and virological responses at 1 and 2 years of ART were similar to those reported in other African settings [4,10,11,22,35,36] and in Western countries [37]. However, more than 35% of patients had a CD4 count <200 cells/ml and were therefore at increased risk of death due to occurrence of opportunistic diseases. In the M12 cohort, undetectable VL was achieved by 75% of the patients, and 89% had a VL <1,000 copies/ml. In Malawi, 87% of the patients receiving a 2 NRTI/1 NNRTI-based regimen had a VL <1,000 copies/ml after a median of 10 months [10]. The DART study conducted in Zimbabwe and Uganda reported that 72% of patients who received AZT/3TC/TDF for a year had undetectable VL [4]. Also, in a teaching hospital of Kampala, undetectable VL was observed in 86% of patients treated with d4T/3TC/NVP for 1 year [7]. Similarly, in a patient cohort in Khayelitsha, South Africa, 70% of patients achieved undetectable VL at 1 year of ART [11]. In our M24 cohort, undetectable VL was achieved by 71% of the patients. Lower virological success rates have been reported in four urban clinics in Senegal, Côte d'Ivoire, Uganda, and Kenya after 2 years of ART (40–69% had a VL <400 copies/ml) [36].

Overall, 7% of patients with virological failure at M12 and 13% at M24 had resistant virus, which is similar to estimates reported for patients treated with 2 NRTI/1 NNRTI-based therapy in east Africa or South-east Asia after more than 9 months of treatment (9–18%) [10,35,38]. This figure might be underestimated if patients with unsuppressed HIV VL (400–999 copies/ml) but not virological failure also had viral mutations conferring resistance to therapy. More conservative estimates of prevalence of resistance would therefore be 18% and 21% of M12 and M24 patients, respectively, on treatment.

Estimates of resistance and virological and immunological failure might also have been underestimated if some of the deaths or patients who were lost to follow-up died as a result of treatment failure and resistance. However, most deaths and losses to follow-up occurred within the first months of therapy when the likelihood of treatment failure was lower. Conversely, because our study is based on outcome data collected at a single time point and no confirmation of laboratory data was obtained in the following weeks, we might have slightly overestimated rates of virological and immunological failure due to measurement errors or to individual temporal variability in biological markers (e.g. HIV viral blips). As in previous studies conducted in patients using primarily d4T/3TC/NVP, the most commonly observed resistance mutations were those associated with the use of NNRTIs (K103N) and 3TC (M184V) [7,10,38], and none of the isolated viruses had mutations conferring resistance to the new NNRTIs (etravirine).

Patients on ART for 2 years were twice as likely as those on 1 year treatment to develop virological failure. Patients diagnosed with TB after ART initiation were also more likely to fail therapy, suggesting that patients' virological response could be impaired when rifampicin-based anti-TB treatment is initiated while a patient is under ART. The potential interaction between NVP and rifampicin in HIV-infected patients treated for TB has been reported in previous studies [39-43]. This can result in subtherapeutic NVP plasma concentrations [39] and favor the development of resistance to this drug. Alternatively, pill burden associated with concomitant treatments could lead to decreased adherence to one or both regimens.

Early identification of patients with virological failure and switch to second-line therapy is important to prevent the accumulation of resistance that might compromise the effectiveness of subsequent lines of treatment. In this program where virological monitoring of patients is not routinely performed, patients presenting general clinical symptoms, those with a weight loss below the value observed at therapy initiation, or those reporting poor adherence were more likely to have virological failure. The association between high VL levels and subtherapeutic NNRTI concentrations has been previously reported in Malawi [44] and Cambodia [38], highlighting the importance of maintaining and reinforcing patients' adherence over time. In our study, patients severely immunosuppressed at the time of the survey were not more likely to have virological failure, although this might change if patients remain in a failing regimen for longer periods of time [38].

The pharmacological assessment showed increased plasmatic levels of NNRTI in 23% of patients on NVP-based therapy and 39% of those on EFV. These figures are higher than what has been described in Western countries, and are likely to be related to the lower weight of our patients at ART initiation [44] compared with European patients [45,46]. In contrast, subtherapeutic levels of NVP and EFV were found in 15% and 2% of the patients, respectively. As in previous studies, the risk of having low NNRTI concentrations in plasma was nearly 4 times higher in patients with poor self-reported adherence [44,47]. We also found a higher risk of subtherapeutic ARV levels in patients who reported digestive disorders at the time of the survey. Vomiting, malabsorption, or misclassification bias (e.g. if patients who reported symptoms were also more likely to report poor adherence) could contribute to explain this finding.

## Conclusion

In conclusion, in this HIV/AIDS care program implemented in a rural African setting using a simplified scale-up approach and free generic ART, survival and retention in care were satisfactory at up to 2 years of therapy, but efforts to increase earlier access to treatment and to improve patient management and support to achieve better immunological and virological outcomes on ART remain necessary to prolong patient survival. Early identification of poor adherence during follow-up, especially in the presence of intolerance to ARV and when TB treatment is provided, should be considered to maximize the duration of first-line therapy.

## Competing interests

The authors declare that they have no competing interests.

## Authors' contributions

LA designed and implemented the study, analyzed and managed data, interpreted results, and wrote the manuscript. GG and CO set up and implemented the study in the field and contributed to the interpretations of results. LP performed statistical analyses. PA contributed to the design, set-up, and implementation of the study. M-LC and CLT performed the virological and pharmacological tests and contributed to the interpretation of the results. SB and DO helped to design the study, interpret the results, and draft the manuscript. MP-R performed statistical analysis, interpreted results, and co-wrote the manuscript. All authors read and approved the final manuscript.

## Pre-publication history

The pre-publication history for this paper can be accessed here:

http://www.biomedcentral.com/1471-2334/9/81/prepub
